# Cladosporium keratitis – a case report and literature review

**DOI:** 10.1186/s12886-015-0092-1

**Published:** 2015-08-19

**Authors:** Steve Chih-Hsuan Cheng, Ying-Yu Lin, Chien-Neng Kuo, Li-Ju Lai

**Affiliations:** Department of Medical Education, Chang Gung Memorial Hospital, No.6, W. Sec., Chia-Pu Rd., Puzi City, Chiayi County 61363 Taiwan; Department of Ophthalmology, Chang Gung Memorial Hospital, No.6, W. Sec., Chia-Pu Rd., Puzi City, Chiayi County 61363 Taiwan; Chang Gung University College of Medicine, No.259, Wenhua 1st Rd., Guishan Township, Taoyuan County 33302 Taiwan

## Abstract

**Background:**

Fungal keratitis is one of the major causes of infectious keratitis in tropical countries. Symptoms of fungal keratitis consist of blurred vision, redness, tearing, photophobia, pain and foreign body sensation. If not treated effectively, it could lead to blindness. Common causes include *Candida spp.*, *Fusarium spp.* and *Aspergillus spp.*. With the limited choices of topical antifungal agents, we were faced with Cladosporium keratitis, a rare cause of fungal keratitis.

**Case presentation:**

A 62-year-old Asian male construction worker came to us with intense ocular pain, injection of the conjunctiva, blurred vision, and foreign body sensation in his left eye. His visual acuity was 20/40 OD and 20/400 OS. Slit-lamp exam revealed a corneal ulcer with feathery margin and Descemet’s membrane folding. The culture yielded *Cladosporium species.*. The patient did not show improvements after applying topical natamycin (5 %), topical amphotericin B (1mg/ml), topical fluconazole (2mg/ml) and oral ketoconazole (200mg). After shifting the medical regimen to voriconazole via topical and systemic routes (1mg/ml and 200mg respectively), the keratitis was controlled.

**Conclusions:**

Fungal keratitis remains a challenge for ophthalmologists as there is no evidence suggesting any particular drug or combination of drugs is more effective than another. A review of common topical antifungal agents was done. Voriconazole could be a good choice for treating corneal infection by *Cladosporium species*.

## Background

Fungal infections occur in countries with warmer climates. Any agent capable of infecting humans is a potential infectious agent. Some common causes include *Candida spp.* (yeast), *Fusarium spp.* (filament) and *Aspergillus spp.* (filament) (Table 1). According to relative incidence reports from India, approximately 45 % of all central corneal ulcers are cause by fungi [1]. *Cladosporium spp.* are rare causes of fungal keratitis. They are Ascomycota fungi that are commonly found on plants. The air borne spores make them extremely abundant in outdoor air. Indoors they can be found on moist surfaces. Though rarely pathogenic to humans, they can be causative agents of pulmonary infections, skin lesions, onychomyocosis and keratitis [2]. Fungi are able to gain access into the corneal stroma via multiple routes [3]. A previous epithelial defect or a penetrating injury could allow fungi to enter through the epithelium. Fungal endophthalmitis could invade from the posterior segment through the Descemet’s membrane. In the case of trabeculectomy, the corneo-scleral meshwork becomes a passage for microorganisms. Once within the cornea, fungi can proliferate and spread through the channels. The proteolytic enzymes and mycotoxins can then cause tissue damage. Predisposing factors of fungal keratitis include ocular trauma, contact lens wear, pre-existing corneal surface disease, underlying systemic disease (e.g., diabetes mellitus) and prolonged use of immunosuppressant and antibiotics [4]. It is usually characterized by stromal inflammation. If left untreated, it could lead to corneal scarring which could ultimately result in blindness [5].

**Table 1:** Causative agents for Fungal Keratitis

Filamentous fungi	
Hyaline fungi	Molds Dematiaceous fungi
Common	Common
*Fusarium spp*	*Curvularia spp*
*Aspergillus spp*	Uncommon
Uncommon	*Bipolaris spp*
*Acremonium spp*	*Exserohilum spp*
*Chrysosporium spp*	*Cladosporium spp*
*Scedosporium spp*	*Lasiodiplodia spp*
	*Alternaria spp*
	*Torula spp*
Yeast	
*Candida spp*	

Natamycin is the only drug approved by the United States Food and Drug Administration for treating fungal keratitis. Reports on Cladosporium corneal infection have been scarce [6]. The patient was refractory to a combination of topical and systemic agents.

After switching to voriconazole, we have successfully treated our patient.

## Case presentation

The patient was a 62-year-old Asian male construction worker who worked in a dusty environment. Debris made up of cement hit his left eye during work on the 22nd of November 2014. He experienced intense, sharp, and constant pain. Blurred vision, red eye and foreign body sensation were the main clinical manifestations. Photophobia, swelling and watery discharge were also noted. He was referred to our hospital from a local medical clinic 4 days after the incident. His integumentary system was intact without signs of fungal infection. According to the patient’s statement, he had hypertension and diabetes mellitus under medical control for years. He was not a user of contact lenses. The patient claimed that he had a fungal keratitis in his left eye 10 years ago after trauma. Natamycin was used for more than 3 months during that episode.

**Fig. 1:**
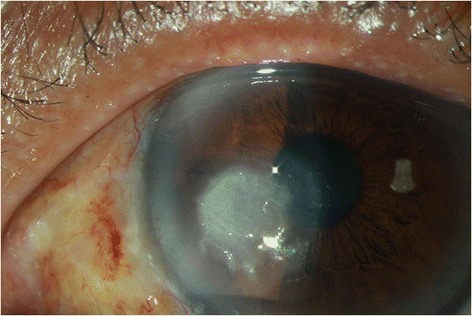
Fungal keratitis caused by *Cladosporium sp.* An external photograph of the left eye of a 62-year-old male construction worker with Cladosporium keratitis. This photograph was taken 4 days after trauma. The corneal ulcer was found with feathery margin and Descemet’s membrane folding. Ring infiltration was also present

**Fig. 2:**
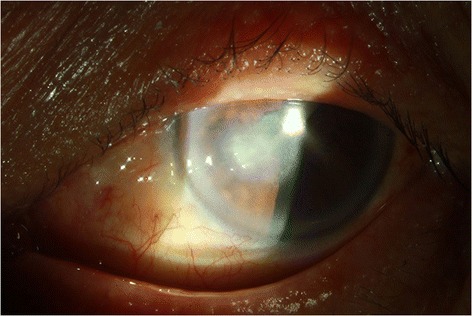
Cladosporium keratitis 3 months after treatment. This external eye photograph was taken from the same patient three months after treatment. The patient’s left eye showed corneal opacity with minimal infiltration

The ocular examination showed his visual acuity to be 20/40 OD and 20/400 OS. The intraocular pressure (IOP) was 15 mmHg OD and 15 mmHg OS. There was a 3×3 mm^2^ epithelial defect with stromal infiltration on the inferior medial area of the left cornea (Fig. 1). The corneal ulcer was found with feathery margin and Descemet’s membrane folding. Ring infiltration was also present. Few fine pigmentary keratic precipitate (KP) and flare were found behind the area of cornea ulcer. The anterior chamber was deep and clear without hypopyon. There was mild nuclear sclerosis of cataract. The vitreous was clear without signs of endophthalmitis. Corneal scraping was done for smear and culture. Under the direct microscopic examination with lactophenol cotton blue (LPCB) wet mount preparation, yeast was present. Repeated cultivations were done on 5 % sheep blood, chocolate, anaerobic blood agar, inhibitory mold agar (IMA), IMA supplemented with chloramphenicol and gentamicin (ICG) agar, and thioglycollate medium. LPCB mount revealed pigmented septate hyphae. Dislodging oval conidia with dark attachment scars characteristic of *Cladosporium sp.* were seen on microscope. Initially the patient was prescribed with natamycin (5 %, QID, Alcon Inc. Texas, USA) and amphotericin B (1mg/mL, Q2H, BMS New York City, U.S. ) for yeast infection and levofloxacin (0.5 %, Q2H, Santen Inc. Japan) for possible concomitant bacterial infection. Topical fluconazole (2mg/mL, Q2H, Pfizer Inc., New York City, U.S.) and oral ketoconazole (200mg/tab, BID, Swiss Co., Taiwan) were then added to the prescription after culture results. Due to the persistent infection, the anti-fungal agent was shifted to voriconazole via topical (1mg/mL, Q2H, Pfizer Inc. New York City, U.S.) and oral routes (200 mg/tab, BID Pfizer Inc. New York City, U.S.) on the 6th day of admission. For better drug penetration, soaking was done on the area of the corneal ulcer with voriconazole for 3 min everyday under local anesthesia. It is then followed by bullous irrigation of Balance Salts Solution (Alcon Inc. Texus, USA) to prevent medicamentosa. The symptoms improved 10 days after admission and the patient was discharged 14 days later. The patient was discharged with topical voriconazole (1mg/mL, QID, Pfizer Inc. New York City, U.S.) for 2 weeks to prevent reactivation. 3 months post-treatment visual acuity was 20/30 OD and 20/40 OS. The IOP was 14 mmHg OD and 14 mmHg OS. Slit lamp biomicroscopy of the left eye showed corneal opacity with minimal infiltration (Fig. 2).

## Conclusion

Leber first documented fungal keratitis in 1879 [7, 8]. Fungal keratitis has preponderance in males with a male to female ratio of 2.25:1 [9]. Molds are far more common than yeast [10]. Patients with ocular trauma were 5.33 times more likely to develop microbial keratitis [11]. The association of trauma was higher for fungal and parasitic keratitis. Out of all fungal keratitis, 81.9 % was caused by trauma [11]. The reason is obvious in that trauma was more often associated with outdoor occupation (e.g. agriculture and manual labor).

Treating fungal keratitis is a laborious process often requiring months.

A literature search using “Fungal keratitis”, “Cladosporium” and “ophthalmic antifungal agents” as keywords in relevant databases (including Medline, Cochrane Library, and PubMed) was performed.

Common agents including their dosages and side effects were gathered and organized (Table 2). Fungal keratitis can be both treated by medical or surgical therapy. The efficacy of medical treatment depends on the penetration of the agent into the aqueous humor and achieving therapeutic levels. Apart from the deep penetration into the cornea by the fungi and the single commercially available antifungal agent (natamycin), resistance to treatment also plays a part. The formation of biofilm is considered to be the cause of resistance [12, 13]. A systemic review of medical interventions for fungal keratitis in the Cochrane Database (updated in 2012) concluded that there is no evidence suggesting that any particular drug or combination of drugs is more effective than another [14]. However, literatures have been emerging to support the use of second generation triazoles, such as voriconazole [6, 15]. Visual acuity and infiltrate size are predictors of worse clinical outcomes. Patients with infiltrates and hypopyon are less likely to respond to medical treatment [16, 17].

**Table 2:** Common topical agents for fungal keratitis

Name	Species	Concentration	Dosing interval	Side effects	Ref.
Polylene drivatives					
Amphotericin B	Aspergillus, spp.	0.15–0.25 %	1st day: Q30mins	Nephrotoxic, bone marrow suppression, anemia, headache	[1]
	Candida spp.		2nd day onward: Q1H		
Natamycin	Fusarium spp.	5 %	5 times a day	Burning, irritation, punctate keratitis, chemosis	[1]
	Aspergillus spp.				
	Candida spp.				
Azole – Imidazole derivatives					
Miconazole	Aspergillus spp.	1 %	Q1H	Punctate epithelial erosions, pruritus, irritation	[2, 3]
	Scedosporium spp.	Ointment: 2 %			
	Candida spp.				
Clotrimazole	Aspergillus spp.	1 %	Q1H	Irritation, punctate keratopathy, hepatotoxic, diarrhea, nausea	[4]
	Fusarium spp.				
Econazole	Aspergillus spp.	2 %	Q4H ~ QID	Local irritation	[5]
	Fusarium spp.				
Ketoconazole	Aspergillus spp.	1 %	QID	Gynecomastia, impotence, hepatotoxic	[6, 7]
	Fusarium spp.				
	Curvularia spp.				
Azole – Triazole derivatives					
Fluconazole	Filamentous fungi	0.2 %	Q4H	Irritation, burning sensation	[8]
Voriconazole	Filamentous fungi	1 %	Q1H	Visual disturbances, skin rash	[9, 10]
	Candida spp.				
Pyramidine drivatives					
Flucytosine	Candidda spp.	1 %	Q1H	Irritation, itching, burning sensation, nausea, vomit, diarrhea	[11]

In a general sense, triazoles were used for yeasts and amphotericin B was used for molds. A good initial treatment would be a combination of natamycin 5 % drops and amphotericin B 0.15 % drops. Additional topical, subconjunctival and or systemic treatment could then be considered depending on the depth and severity of the infection and the culture result. The treatment regimen should be adjusted according to the clinical progression based on biomicroscopic signs, repeated corneal scrapings and tolerance of medications. It is also important to put the patient’s compliance into account.

Amphotericin B alters the stability of the membrane by binding to ergosterol and forms pores. It is insufficiently absorbed from the GI tract and due to its poor ocular penetration, administration of higher doses via the intravenous route is needed [4]. Amphotericin also binds to mammalian cholesterol albeit with lower affinity. Thus explaining its side effects. It causes chills and fever and it is notorious for its nephrotoxicity [4]. It is the drug of choice for Candida keratitis. Though also effective against filamentous fungi, it has no activity against *Fusarium sp*..

Natamycin is the only ophthalmic agent approved by the Food and Drug Administration. It also binds to ergosterol. However, it does not alter the membrane permeability. In stead, it prevents the ergosterol-dependent fusion of vacuoles and membrane fusion and fission [18]. It has good activity against *Candida*, *Aspergillus* and *Fusarium spp.* Though being used as a standard care, the penetration is poor and the bioavailablity is only about 2 % after topical administration [19]. It is therefore not the drug of choice for deep, severe infection.

Corticosteroids (imidazoles or triazoles) inhibit sterol demethylation of lanosterol to ergosterol in fungal membranes. Fluconazole is a safe agent that can be administered orally, intravenously, subconjunctivally or topically. The penetration is well with few side effects [20]. It is limited by its narrow spectrum of antifungal activity. It is inactive against *Aspergillus* and *Fusarium spp*. Miconazole has a broad spectrum of activity however, it is toxic systematically and could lead to epithelial erosions topically. It is used as a second-line agent to natamycin [21].

Voriconazole was first tested for retinal toxicity in rodent animal models [22]. No electroretinographic or histological abnormality was reported with an intravitreal voriconazole concentrations up to 25 μg/mL. Voriconazole has an excellent susceptibility profile against both yeasts and molds. It does not depend on the state of the epithelial surface [23]. The ocular penetration of voriconazole after two 400mg doses of voriconazole 12 h apart was measured at 1.13 μg/mL (53 % of plasma levels) [24]. The safety profile of voriconazole has been reviewed [25]. Visual disturbances including photophobia and/or color change and skin rashes were mild and transient. Even with continued therapy, they typically resolve within 1 month. Oral and IV formulations were approved by FDA for deadly fungal infections in 2002. The broad spectrum of antifungal activity includes species that are resistant to other antifungal agents [24]. In the high-risk group population for developing fungal keratitis or endophthalmitis, voriconazole can also be used as a prophylactic agent [24].

Despite the recent advancement in diagnosis and treatment of fungal keratitis, 15–27 % require surgery [26]. Surgical mode of treatment includes debridement, penetrating keratoplasty, evisceration, bandage contact lens and corneal transplantation. Surgical intervention is carried out in a significantly larger number of patients with fungal keratitis compared to bacterial and parasitic keratitis thus indicating the poor response of fungal keratitis to medical treatment [11].

In conclusion, voriconazole could be a good choice for refractory fungal keratitis. We were able to control the Cladosporium keratitis by combining oral and topical voriconazole.

## Consent

Written informed consent was obtained from the patient for publication of this Case report and any accompanying images. A copy of the consent form is available for review by the Editor of this journal.
